# Whole-Genome Sequences of Two Closely Related Bacteria, *Actinomyces* sp. Strain Chiba101 and *Actinomyces denticolens* DSM 20671^T^

**DOI:** 10.1128/genomeA.00126-17

**Published:** 2017-04-06

**Authors:** Yu Kanesaki, Taichiro Ishige, Yuriko Sekigawa, Tomoko Kobayashi, Yasushi Torii, Eiji Yokoyama, Hiroyuki Ishiwata, Moriyuki Hamada, Tomohiko Tamura, Ryozo Azuma, Satoshi Murakami

**Affiliations:** a NODAI Genome Research Center, Tokyo University of Agriculture, Tokyo, Japan; b Laboratory of Animal Health, Department of Animal Science, Tokyo University of Agriculture, Funako, Atsugi, Kanagawa, Japan; c Chiba Prefectural Institute of Public Health, Nitona, Chuo, Chiba, Japan; d Technical Research Institute, Nishimatsu Construction Co. Ltd., Shinbashi, Minato-ku, Tokyo, Japan; e Biological Resource Center, National Institute of Technology and Evaluation (NBRC), Kisarazu, Chiba, Japan; f Department of Animal Science, Tokyo University of Agriculture, Funako, Atsugi, Kanagawa, Japan

## Abstract

*Actinomyces* sp. strain Chiba101, isolated from an arthritic leg joint of a pig raised in Japan, is a bacterium closely related to* Actinomyces denticolens*. Here, we deciphered the complete genome sequence of *Actinomyces* sp. Chiba101 and the high-quality draft genome sequence of *A. denticolens* DSM 20671^T^.

## GENOME ANNOUNCEMENT

Cells of members of the genus *Actinomyces* are straight or slightly curved rods to filament with true branching. They are Gram-positive and facultative anaerobic actinobacteria, and are associated with a variety of infections in various animals. Several strains that were similar to “*Actinomyces suis*” Franke 1973 had been isolated from the tonsils, mammary gland, and an arthritic leg joint in pigs raised in Japan, and the representative strain was designated Chiba101 (1). These isolates have been found to be pathogenic in pigs (2, 3). However, since no type strain of *A. suis* Franke 1973 was extant (4), it is impossible to compare strain Chiba101 and *A. suis* Franke 1973. On the other hand, *Actinomyces denticolens* has been known as a commensal bacterium found in the oral cavity of cattle (5). Interestingly, we also found that strain Chiba101 had chemotaxonomical and serological characteristics which were identical to those of *A. denticolens*. Therefore, we carried out whole-genome sequencing of strain Chiba101 and *A. denticolens* DSM 20671^T^ in order to clarify the taxonomic relationship between strain Chiba101 and *A. denticolens*.

Genomic DNA was extracted from a single colony from each strain. DNA detection was carried out using the Dr. GenTLE High Recovery (TaKaRa Bio, Inc., Kyoto, Japan), according to the manufacturer’s protocol. DNA libraries were sequenced through the massively parallel sequencing method using a PacBio RSII (Pacific Biosciences of California, Inc.), as described by the manufacturer. Approximately 380 Mbp and 113 Mbp of sequencing data were obtained for strains Chiba101 and *A. denticolens* DSM 20671^T^, respectively. Sequence reads from the PacBio RSII were assembled using Celera Assembler version 7.0. Only one supercontig was assembled for strain Chiba101. After checking the overhanging region at both ends, the supercontig can be circularized, with a total length of 2,877,864 bp in the case of strain Chiba101, with a G+C content of 71.28%. In the case of *A. denticolens* DSM 20671^T^, the eight contigs (>1 kb) were assembled with a total length of 2,904,857 bp, with a G+C content of 71.23%. An annotation of the complete genome of strain Chiba101 and the eight contigs of *A. denticolens* DSM 20671^T^ was performed using the Microbial Genome Annotation Pipeline (MiGAP) (6), as well as manual curation. As a result, nine rRNAs, 51 tRNAs, and 2,364 coding sequences (CDSs) were identified for strain Chiba101. For *A. denticolens* DSM 20671^T^, 14 rRNAs, 51 tRNAs, and 2,510 CDSs were identified.

Our sequencing data will largely contribute to future studies for elucidating not only the taxonomic position of strain Chiba101 but also the epidemiological features and genetic variation among strains of *A. denticolens*.

### Accession number(s).

The complete genome sequence of *Actinomyces* sp. Chiba101 (accession number AP017896) and draft genome sequence of *A. denticolens* DSM 20671^T^ (accession numbers BDIO01000001 to BDIO01000008) have been deposited in DDBJ/ENA/GenBank.
